# Nivolumab after recurrent pembrolizumab-associated hepatotoxicity in PD-L1-high metastatic squamous non-small cell lung cancer: a case report

**DOI:** 10.1093/omcr/omag159

**Published:** 2026-08-22

**Authors:** Adam Bowen, Katlyn Wendel, Muhammad-Danish Saleem, Zijin Lin, Ramalakshmi Thulluri, Borys Hrinczenko

**Affiliations:** Department of Internal Medicine, McLaren Greater Lansing Hospital, Michigan State University, 2900 Collins Rd, Lansing, MI 48910, United States; Department of Internal Medicine, McLaren Greater Lansing Hospital, Michigan State University, 2900 Collins Rd, Lansing, MI 48910, United States; Department of Hematology/Oncology, Karmanos Cancer Institute at McLaren Greater Lansing Hospital, Michigan State University, 3520 Forest Rd, Lansing, MI 48910, United States; Department of Internal Medicine, McLaren Greater Lansing Hospital, Michigan State University, 2900 Collins Rd, Lansing, MI 48910, United States; Department of Internal Medicine, McLaren Greater Lansing Hospital, Michigan State University, 2900 Collins Rd, Lansing, MI 48910, United States; Department of Hematology/Oncology, Karmanos Cancer Institute at McLaren Greater Lansing Hospital, Michigan State University, 3520 Forest Rd, Lansing, MI 48910, United States

**Keywords:** non-small cell lung cancer, pembrolizumab, nivolumab, immune-related hepatitis, retreatment

## Abstract

Immune-related hepatitis can interrupt effective PD-1 therapy, and evidence guiding within-class retreatment after recurrent toxicity remains limited. An 82-year-old woman with remote resected lung cancer later developed PD-L1-high metastatic squamous non-small cell lung cancer with thoracic and hepatic disease. After 4 pembrolizumab doses, mixed liver injury developed despite normal baseline liver chemistries. Viral hepatitis serologies were negative, liver ultrasound was unrevealing, and acetaminophen exposure remained a non-significant competing factor. Liver tests improved with corticosteroids but recurred after 2 pembrolizumab rechallenges, including a grade 3 event, prompting permanent discontinuation. Nivolumab was started after biochemical recovery and was tolerated for more than 3 years without recurrent hepatotoxicity. Follow-up PET-CT demonstrated durable metabolic resolution of thoracic and hepatic disease.

## Introduction

Pembrolizumab plus carboplatin and paclitaxel or nab-paclitaxel is established first-line therapy for metastatic squamous non-small cell lung cancer and improved survival in KEYNOTE-407 [1]. Immune checkpoint inhibitors cause a distinct spectrum of immune-related adverse events, and hepatic toxicity is reported in approximately 1%–6% of patients receiving PD-1/PD-L1 blockade, with grade 3–4 events in roughly 1%–3% [2, 3]. Anti-PD-1 hepatitis may be hepatocellular, mixed, or cholestatic, and management requires exclusion of alternative causes together with corticosteroids when abnormalities are persistent or clinically significant [4–8]. Data supporting retreatment after recurrent hepatitis remain limited, particularly for switching from pembrolizumab to nivolumab after repeated same-agent rechallenge failure [9, 10].

## Case report

**Figure 1:**
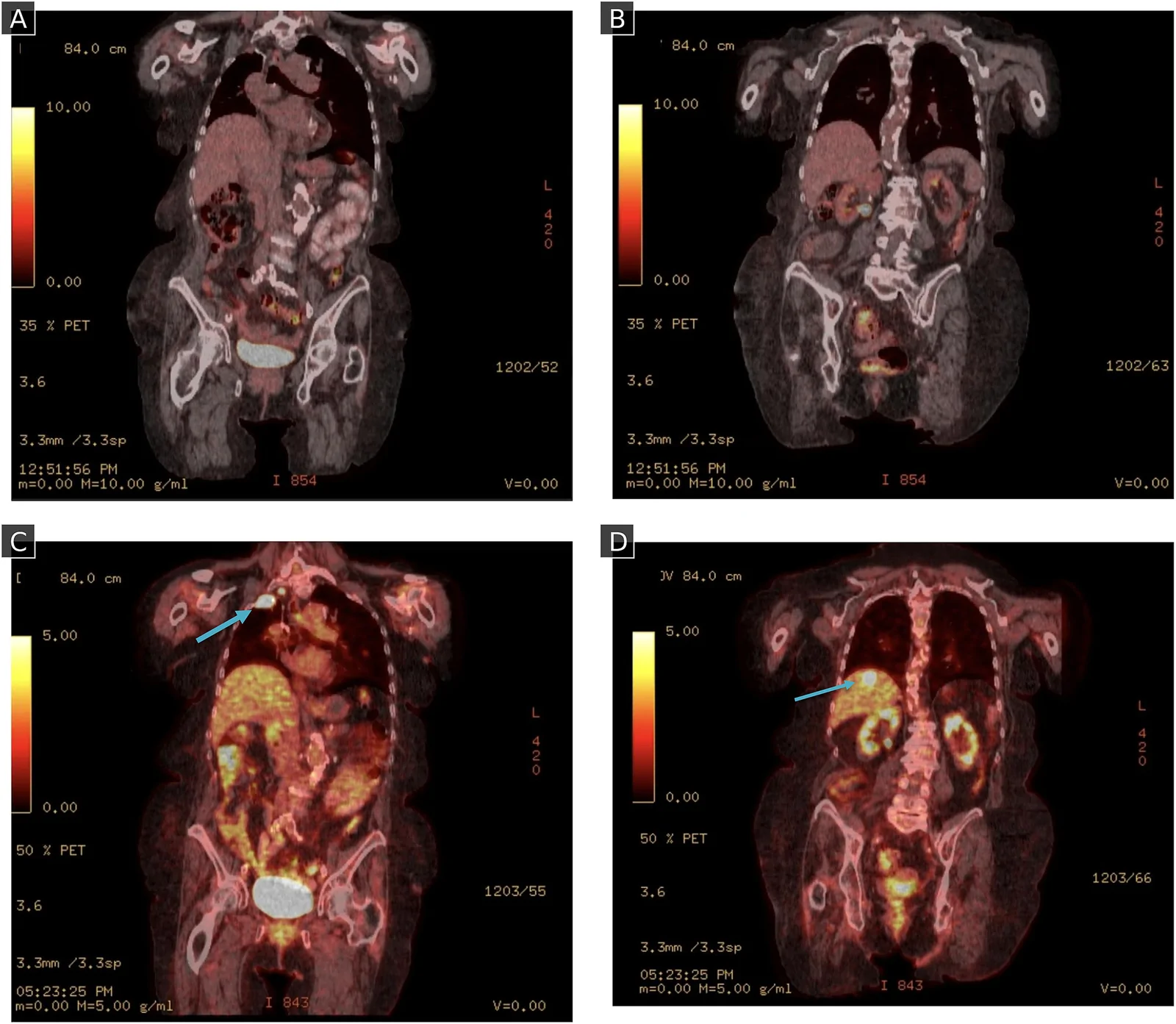
Representative fused coronal PET-CT images before and after nivolumab. Panels C and D are baseline images obtained before pembrolizumab, demonstrating the right apical lung lesion with nodal disease and multiple hypermetabolic right hepatic lesions. Panels A and B are post-nivolumab images demonstrating marked interval metabolic response with resolution of the previously described thoracic and hepatic hypermetabolic disease. Arrows point to the hypermetabolic liver and apical lung lesions.

An 82-year-old woman had undergone right upper lobectomy and adjuvant chemotherapy for stage II large cell carcinoma more than a decade earlier. Seven weeks before pembrolizumab, PET-CT demonstrated a 3 cm hypermetabolic right apical mass (SUV 17.7), right retroclavicular and right paratracheal nodal disease, additional left-sided pulmonary nodules, at least 3 hypermetabolic right hepatic lesions, and a mesenteric nodule (Fig. 1C and D). Ultrasound-guided biopsy of a liver lesion 4 weeks before pembrolizumab showed poorly differentiated carcinoma consistent with metastatic squamous cell carcinoma, strongly CK5/6 positive, focally weak p40/p63 positive, and TTF-1 negative. Prior pathology slides were unavailable, so the later malignancy was managed clinically as PD-L1-high metastatic squamous non-small cell lung cancer, with recurrence versus metachronous second primary unresolved. Carboplatin/paclitaxel were started 3 weeks before pembrolizumab, and pembrolizumab 200 mg every 3 weeks was added because tumour proportion score was 100%, consistent with a KEYNOTE-407-supported chemoimmunotherapy strategy [1]. The relative chronology is summarized in Table 1.

**Table 1:** Relative chronology of diagnosis, treatment, liver injury, and outcome.

**Relative time**	**Key event**	**Selected findings**	**Management / outcome**
More than 10 years before pembrolizumab	Remote prior lung cancer	Stage II large cell carcinoma treated with right upper lobectomy and adjuvant chemotherapy.	Original pathology unavailable, leaving recurrence versus metachronous second primary unresolved.
7 weeks before pembrolizumab	Baseline PET-CT	3 cm hypermetabolic right apical mass, metastatic nodal disease, left-sided pulmonary nodules, at least 3 hypermetabolic hepatic lesions, and mesenteric nodule.	Established metastatic disease burden before immunotherapy.
4 weeks before pembrolizumab	Liver biopsy and baseline chemistry	Biopsy consistent with metastatic squamous cell carcinoma, CK5/6 strong positive, focal weak p40/p63 positive, TTF-1 negative. Baseline AST 19, ALT 21, ALP 71, bilirubin 0.5.	CARIS profiling showed PD-L1 TPS 100%.
3 weeks before pembrolizumab	Induction chemotherapy	Carboplatin AUC 6 plus paclitaxel 175 mg/m2 started.	Systemic treatment begun.
Week 0	Pembrolizumab initiation	Pembrolizumab 200 mg every 3 weeks added.	Continued because tumour proportion score was 100%.
Week 12	First documented liver injury	AST 134, ALT 194, ALP 217, bilirubin 0.7.	Pembrolizumab held, competing causes evaluated.
Weeks 13-15	Persistent early flare	AST 76-84, ALT 130-131, ALP 303-333, bilirubin 0.5-0.6.	Hepatitis A IgM, hepatitis B core IgM, hepatitis B surface antigen, and hepatitis C antibody negative. Liver ultrasound unrevealing.
Weeks 15-18	Prednisone course and first rechallenge	Prednisone started, then pembrolizumab resumed after improvement.	Acetaminophen exposure for ankle pain remained a competing but decreasing factor.
Week 21.6	Recurrent grade 3 event	AST 214, ALT 263, ALP 429, bilirubin 1.3.	Pembrolizumab held again.
Weeks 24-25	Persistent cholestatic-predominant flare	AST 156, ALT 230, ALP 622, bilirubin 0.9.	Additional prednisone prescribed.
Weeks 30-33	Further recurrence before switch	AST 98-152, ALT 90-159, ALP 209-449, bilirubin 0.6-1.1.	Prednisone prescribed again, pembrolizumab permanently discontinued.
Week 33.7	Nivolumab initiation	Nivolumab 480 mg every 4 weeks started after improvement to grade 1 or better.	No recurrent hepatotoxicity thereafter.
Week 87.6	Response PET-CT	Interval resolution of right apical mass, nodal disease, and hepatic metastases.	No new hypermetabolic lesions.
Weeks 120-172	Later surveillance PET-CTs	No progressive hypermetabolic disease.	Durable response while on nivolumab.
Week 207	Death	Date of death documented as 4/5/2024.	Cause of death unclear.

Abbreviations: ALP, alkaline phosphatase, PET-CT, positron emission tomography-computed tomography, TPS, tumour proportion score.

**Figure 2:**
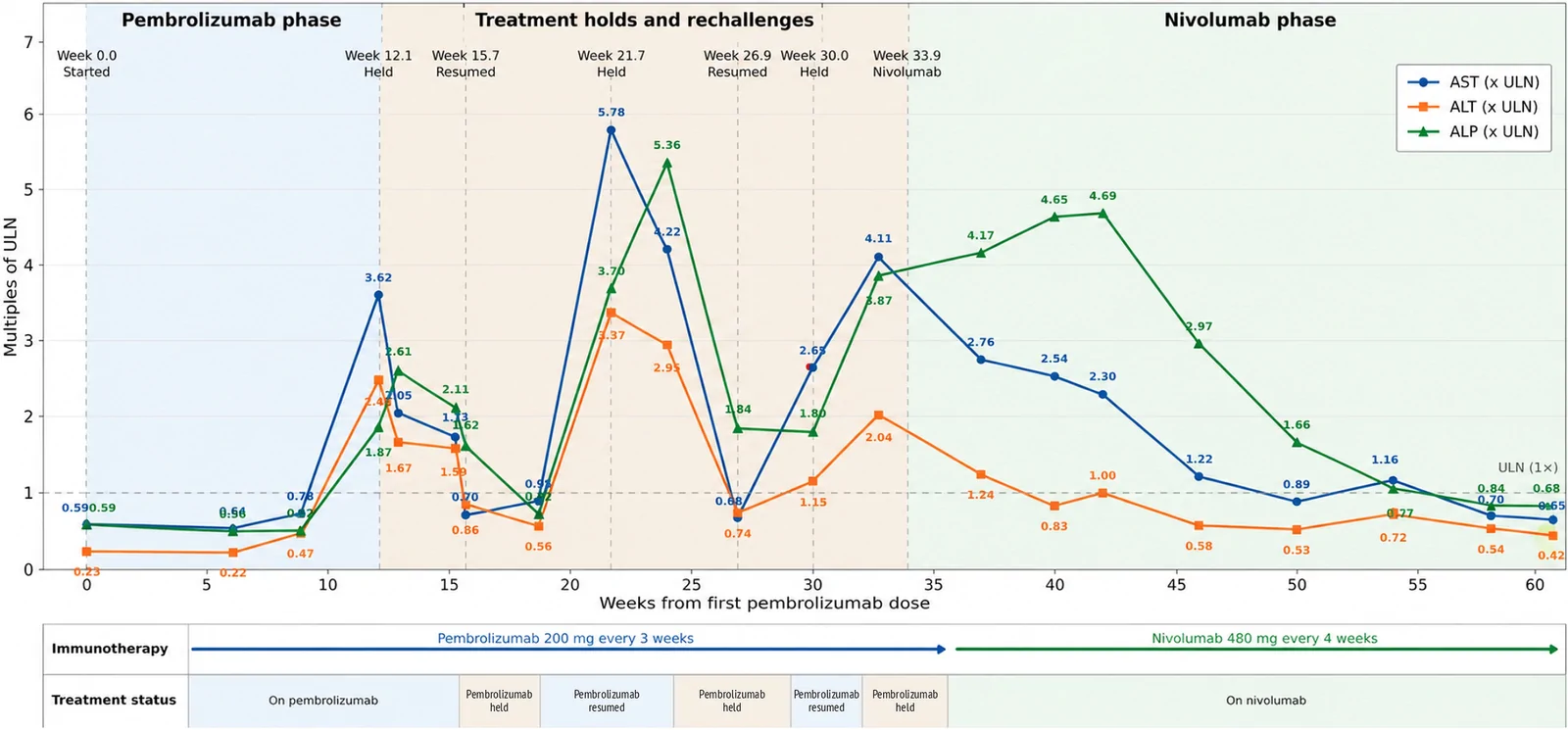
Relative course of liver chemistries and key treatment phases, anchored to the first pembrolizumab dose (week 0). AST, ALT, and alkaline phosphatase are plotted as multiples of the local upper limit of normal. Shaded bands denote pembrolizumab exposure, documented prednisone tapers, and nivolumab maintenance. Vertical markers identify the first pembrolizumab hold, rechallenge, grade 3 recurrence, and nivolumab initiation.

Pretreatment liver chemistries were normal: AST 19 [<34], ALT 21 [10–49], alkaline phosphatase 71 [40–116], and total bilirubin 0.5 [0.2–1.2]. After 4 pembrolizumab doses, around week 12, mixed liver injury developed with AST 134 [<34], ALT 194 [10–49], alkaline phosphatase 217 [40–116], and bilirubin 0.7 [0.2–1.2]. Repeat testing around weeks 13–15 remained abnormal, with alkaline phosphatase rising to 333 [40–116]. Viral hepatitis serologies, including hepatitis A IgM, hepatitis B core IgM, hepatitis B surface antigen, and hepatitis C antibody, were negative, and liver ultrasound was unrevealing. Notably the patient also had acetaminophen (Tylenol) use for ankle pain during this early transaminitis episode, reported as 325 mg every 4 hours, equivalent to 1950 mg/day, with duration not documented in available records, and serum acetaminophen level was not assessed. The patient was counseled to discontinue and limit usage after transaminitis was noted. Liver biopsy was deferred during toxicity because liver chemistries improved after pembrolizumab interruption and corticosteroids, serologies and ultrasound did not identify an alternative process, and biopsy results were not expected to change acute management. Because hepatic metastases commonly explain abnormal liver tests during pembrolizumab therapy, alternative attribution remained clinically important [8]. Selected liver chemistries are shown in Fig. 2.

Pembrolizumab was held and prednisone was prescribed at approximately weeks 15–18, including an initial 50 mg course followed by a taper. After improvement, pembrolizumab was rechallenged twice. Recurrent liver injury followed each re-exposure. At week 21, AST rose to 214 [<34], ALT to 263 [10–49], alkaline phosphatase to 429 [40–116], and bilirubin to 1.3 [0.2–1.2]. A further flare around weeks 24–25 showed AST 156 [<34], ALT 230 [10–49], alkaline phosphatase 622 [40–116], and bilirubin 0.9 [0.2–1.2]. Additional prednisone tapers were prescribed at approximately weeks 24–27 and again at week 33, after which pembrolizumab was permanently discontinued.

Because alternative systemic options were limited, biochemical recovery to grade 1 or better had occurred, and therapeutic need remained high, nivolumab 480 mg every 4 weeks was initiated at week 33 after an individualized risk–benefit discussion acknowledging that guideline-based management would generally favor permanent discontinuation of immune checkpoint therapy after recurrent or grade 3–4 immune-related hepatitis [6, 7]. No recurrent hepatotoxicity occurred. Liver chemistries normalized over subsequent months during nivolumab. PET-CT at week 87.6 demonstrated interval resolution of the right apical mass, right supraclavicular nodal disease, and hepatic metastases, without new hypermetabolic lesions (Fig. 1A and B). The patient remained on nivolumab for more than 3 years with persistently normal or near-normal liver tests. She died at week 207, from suspected cardiac arrest at home.

## Discussion

Viral hepatitis was excluded serologically, but hepatic metastases were present from diagnosis and acetaminophen exposure was documented during an early episode. Such competing causes are common and, in some series, explain abnormal liver tests more often than true pembrolizumab hepatotoxicity [8]. Even so, the overall pattern favored pembrolizumab-associated liver injury: temporal association with exposure, improvement after interruption and corticosteroids, reproducible recurrence on rechallenge, and prolonged absence of recurrence after switching to nivolumab. Dechallenge and rechallenge patterns remain among the most practical clinical clues when liver biopsy during toxicity is unavailable [4]. Biopsy was further limited by age 82, multiple hepatic metastases, potential bleeding or sampling error, and patient preference against an invasive procedure.

The biochemical pattern was mixed and cholestatic-predominant rather than purely hepatocellular, because alkaline phosphatase rose disproportionately during recurrent episodes. That is compatible with the broader spectrum reported for anti-PD-1 liver injury [2–5]. ASCO and ESMO guidance supports withholding immune checkpoint therapy for grade 2 hepatitis, using corticosteroids when clinically indicated, and permanently discontinuing immune checkpoint therapy for recurrent or severe grade 3–4 toxicity [6, 7]. Nivolumab was therefore an individualized exception after biochemical recovery, informed consent, limited alternatives, and planned close monitoring. Retreatment after checkpoint inhibitor hepatitis can be feasible in selected patients, but published data largely pool tumour types and retreatment strategies, and direct evidence for pembrolizumab-to-nivolumab substitution after repeated pembrolizumab rechallenge failure is sparse [9, 10].

This report does not establish class-wide safety or contradict guideline recommendations for permanent discontinuation after recurrent or grade 3–4 hepatitis. Instead, it supports a narrower conclusion: after careful assessment of competing causes, biochemical recovery, and informed risk–benefit discussion, within-class PD-1 substitution may remain reasonable only as an individualized exception when therapeutic need is high and close laboratory monitoring is feasible [4, 6, 7, 9, 10]. Principal limitations are lack of liver histology during toxicity, which was not pursued because clinical improvement made biopsy unlikely to alter acute management, unresolved classification of recurrence versus second primary, and unclear documented cause of death.
